# Compositional analysis of the associations between 24-h movement behaviours and cardio-metabolic risk factors in overweight and obese adults with pre-diabetes from the PREVIEW study: cross-sectional baseline analysis

**DOI:** 10.1186/s12966-020-00936-5

**Published:** 2020-03-04

**Authors:** Nils Swindell, Paul Rees, Mikael Fogelholm, Mathijs Drummen, Ian MacDonald, J. Alfredo Martinez, Santiago Navas-Carretero, Teodora Handjieva-Darlenska, Nadka Boyadjieva, Georgi Bogdanov, Sally D. Poppitt, Nicholas Gant, Marta P. Silvestre, Jennie Brand-Miller, Wolfgang Schlicht, Roslyn Muirhead, Shannon Brodie, Heikki Tikkanen, Elli Jalo, Margriet Westerterp-Plantenga, Tanja Adam, Pia Siig Vestentoft, Thomas M. Larsen, Anne Raben, Gareth Stratton

**Affiliations:** 1 Engineering East, Swansea University, Fabian Way, Crymlyn Burrows, Skewen, Swansea, Wales SA1 8EN; 2 University of Helsinki, Helsinki, Finland; 3 Maastricht University, Maastricht, Netherlands; 4 University of Nottingham, Nottingham, UK; 5 Centre for Nutrition Research, University of Navarra (UNAV), Pamplona, Spain; 6 CIBERObn, Instituto de Salud Carlos III, Madrid, Spain; 7 IdiSNA, Navarra Institute for Health Research, Pamplona, Spain; 8 Program for Precision Nutrition, IMDEA Food Institute, Madrid, Spain; 9 Medical University of Sofia, Sofia, Bulgaria; 10 Human Nutrition Unit, School of Biological Sciences, University of Auckland, Auckland, New Zealand; 11 University of Sydney, Sydney, Australia; 12 University of Stuttgart, Stuttgart, Germany; 13 University of Eastern Finland, Kuopio, Finland; 14 University of Copenhagen, Copenhagen, Denmark

**Keywords:** Physical activity, Sedentary time, Compositional analysis, Pre-diabetes

## Abstract

**Background:**

Physical activity, sedentary time and sleep have been shown to be associated with cardio-metabolic health. However, these associations are typically studied in isolation or without accounting for the effect of all movement behaviours and the constrained nature of data that comprise a finite whole such as a 24 h day. The aim of this study was to examine the associations between the composition of daily movement behaviours (including sleep, sedentary time (ST), light intensity physical activity (LIPA) and moderate-to-vigorous activity (MVPA)) and cardio-metabolic health, in a cross-sectional analysis of adults with pre-diabetes. Further, we quantified the predicted differences following reallocation of time between behaviours.

**Methods:**

Accelerometers were used to quantify daily movement behaviours in 1462 adults from eight countries with a body mass index (BMI) ≥25 kg**·**m^− 2^, impaired fasting glucose (IFG; 5.6–6.9 mmol**·**l^− 1^) and/or impaired glucose tolerance (IGT; 7.8–11.0 mmol•l^− 1^ 2 h following oral glucose tolerance test, OGTT). Compositional isotemporal substitution was used to estimate the association of reallocating time between behaviours.

**Results:**

Replacing MVPA with any other behaviour around the mean composition was associated with a poorer cardio-metabolic risk profile. Conversely, when MVPA was increased, the relationships with cardiometabolic risk markers was favourable but with smaller predicted changes than when MVPA was replaced. Further, substituting ST with LIPA predicted improvements in cardio-metabolic risk markers, most notably insulin and HOMA-IR.

**Conclusions:**

This is the first study to use compositional analysis of the 24 h movement composition in adults with overweight/obesity and pre-diabetes. These findings build on previous literature that suggest replacing ST with LIPA may produce metabolic benefits that contribute to the prevention and management of type 2 diabetes. Furthermore, the asymmetry in the predicted change in risk markers following the reallocation of time to/from MVPA highlights the importance of maintaining existing levels of MVPA.

**Trial registration:**

ClinicalTrials.gov (NCT01777893).

## Introduction

Higher levels of MVPA are associated with markers of better cardio-metabolic health, lower incidence of type 2 diabetes and lower all-cause-mortality [1]. Although physical activity research has typically focused on MVPA, emerging evidence suggests that light intensity physical activity (LIPA) is associated with better cardio-metabolic health, lower incidence of type 2 diabetes and all-cause mortality after adjusting for MVPA [2–4]. On the other hand, sedentary time is adversely associated with cardio-metabolic health [5, 6], incidence of type 2 diabetes, cardiovascular disease [7] and all-cause mortality [8] independent of MVPA. Additionally, there is some evidence that both short and long sleep duration are adversely associated with body mass index (BMI), impaired glucose metabolism and blood pressure [9].

A limitation to previous literature is that associations between health outcomes and time allocated to these behaviours have typically been studied in isolation with only partial adjustment for time spent in other behaviours [10]. Because the duration of a day (24 h) is fixed, the subcomponents of the day (in this case the behavioural domains of sleep, ST, LIPA and MVPA) can be considered as relative contributions to the whole. Time spent in one behaviour necessarily displaces time spent in, at least one of the remaining behavioural domains. Consequently, the complete set of behavioural domains are perfectly collinear and cannot be used in traditional multivariate analyses [11, 12]. Such data, comprising of mutually exclusive parts of a whole, are inherently compositional in nature and should be analysed with this in mind [11, 12]. Isotemporal substitution models developed by Makery and colleagues [13] address the constrained nature of 24-h time use data. This approach uses linear models to explore the theoretical effect of reallocating time between behaviours. However, these models treat units of time as absolute measures and therefore do not account for the relative nature of time-use data. In contrast, compositional analysis recognises the constrained nature of such data and uses log ratios to express the composition in terms of ratios of its parts. Conveying compositional data as log ratio coordinates transforms them from the constrained simplex to the unconstrained real space in which traditional multi variate statistics can be applied [12, 14, 15]. The shift towards compositional analysis in physical activity research was pioneered by Chastin et al. [11], Carson et al. [16], and Dumuid et al. [4, 15] and allows the examination of the combined effect of the activity composition on indicators of health. Furthermore, compositional isotemporal substitution can estimate change in health indicators following the reallocation of time between behaviours.

There is a growing body of evidence supporting the use of compositional data analysis (CoDa) in studies of cardiometabolic risk markers and obesity in both adults [11, 17] and children [4, 16, 18]. In a sample of adults at high risk of type 2 diabetes, Biddle and colleagues [19] found that stepping time was associated with markers of metabolic health relative to sleep, sitting and standing. To our knowledge, no studies have used compositional isotemporal substitution among behavioural domains sleep, ST, LIPA and MVPA in adults with pre-diabetes.

Therefore, the aim of this study was to investigate the reallocation of time between behaviours (sleep, ST, LIPA and MVPA) and their associations with cardio-metabolic risk markers using compositional isotemporal substitution in a large international sample of overweight and obese adults with pre-diabetes.

## Methods

### Participants and setting

This study is a cross-sectional analysis of the baseline data from the PREVention of diabetes through lifestyle Intervention and population studies in Europe and around the World (PREVIEW) study. PREVIEW is registered with ClinicalTrials.gov (NCT01777893) and a detailed protocol has been published elsewhere [20]. Briefly, the PREVIEW study is a large diabetes prevention intervention conducted at eight study sites: University of Copenhagen (Denmark), University of Helsinki (Finland), University of Maastricht (The Netherlands), University of Nottingham (UK), University of Navarra (Spain), Medical University of Sofia (Bulgaria), University of Sydney (Australia) and the University of Auckland (New Zealand).

A detailed description of the recruitment and screening process has been published previously [20]. Briefly, participants were recruited using varied methods across the 8 study sites which included newspaper, radio and television advertisements and direct contact with primary and occupational health care providers. Interested individuals were pre-screened for eligibility using the Finnish Diabetes Risk Score [21] before attending a laboratory screening. Following the laboratory screening, 2326 participants met the inclusion criteria: age 25–70 years; BMI > 25 kg/m^2^; pre-diabetes confirmed at oral glucose tolerance test (OGTT). Pre-diabetes was defined in line with ADA criteria [22], as either (i) increased fasting glucose (IFG), with venous plasma glucose concentration of 5.6–6.9 mmol**·**l^− 1^ and/or (ii) impaired glucose tolerance (IGT), with venous plasma glucose concentration of 7.8–11.0 mmol**·**l^− 1^ at 2 h and fasting plasma glucose < 7.0 mmol**·**l^− 1^. All participants were free of any illness and/or medication that had the potential to affect the compliance or outcomes of the study. Of the 2326 eligible participants, 1699 had 4 days and 3 nights of valid accelerometer data, 237 were identified with acute inflammation (hs-CRP > 10 mg**·**l^− 1^) and removed. Finally, 1462 were included in the analysis.

### Measurements and procedures

Data was collected at the baseline prior to any dietary intervention or weight loss*.*

#### Physical activity, sedentary time and sleep

Participants wore an ActiSleep+ (ActiGraph LLC, Pensacola, FL) accelerometer on the right hip for 24 h·day^− 1^ for 7 consecutive days prior to or preceding a clinical examination. Data was collected using 60-s epochs and non-wear was classified as 60-min of consecutive zeros with the allowance of interruptions for up to 2 min [23]. After the removal of nocturnal sleep episodes [24], participants providing at least 4 days, including at least 1 weekend day, of valid data (≥10 h·day^− 1^ of waking wear time) were included in the analysis [25]. Sleep time was determined using a fully-automated algorithm developed for use with 24-h waist-worn accelerometer protocols in children [24] and recently validated in adults [26]. Troiano cut points [23] were used to determine time (minutes·day^− 1^) spent in sedentary, light and moderate-to-vigorous physical activity (MVPA).

Body mass, stature and waist circumference (WC) were measured according to techniques outlined by Lohman et al. [27]. Systolic and diastolic blood pressure were measured to the nearest 1 mmHg using a validated automatic device on the right arm after resting for 5–10 min. Measurements were performed 3 times with a 1-min rest between recordings and the mean value was recorded.

Blood was drawn from the vein in the antecubital fossa after fasting (> 10 h). Blood samples were stored locally at -80 °C, before shipping to the National Institution for Health and Welfare in Helsinki, Finland where they were analysed for glucose, insulin, HbA_1c_, high sensitivity C-reactive protein (hs-CRP), total cholesterol, triglycerides and HDL-cholesterol (HDL-C) concentrations. Insulin resistance was calculated using the homeostasis model assessment for insulin resistance (HOMA-IR), using the equation: HOMA-IR = Fasting insulin (mU·l^− 1^) x Fasting glucose (mmol·l^− 1^) / 22.5. HOMA-IR has been validated against the gold standard hyperinsulinemic-euglycemic clamp technique [28]. LDL-cholesterol (LDL-C) was calculated using Friedewald’s formula [29]. Body fat % was assessed by dual energy X-ray absorptiometry (DXA), bioelectrical impedance (BIA) or Bodpod (details listed in Supplementary Material). Socio-economic variables, including ethnicity, educational status, household income were assessed with self-administered questionnaires [30].

### Statistical analysis

Descriptive statistics (mean ± SD) were calculated for continuous variables and frequencies (%) for categorical variables. Analysis was performed in R (http://cran.r-project.org) using the compositions package (van den Boogaart and Tolosana-Delgado 2008). As an alternative to the arithmetic mean, the compositional mean was computed by, firstly, calculating the geometric mean for each behaviour separately (Sleep, ST, LPA and MVPA) and then normalizing the data to the same constant, in this case 1, to represent proportions of a whole i.e. 24 h [14]. The dispersion of compositional data was estimated using the variation matrix of logs of all possible pair-wise ratios between behaviours [12, 14]. A value close to zero implies that the two parts in the ratio are highly proportional (co-dependent) [14]. All accelerometer variables (sleep, ST, LIPA and MVPA) were expressed as three isometric log-ratio co-ordinates [12, 14].

The isometric log-ratio co-ordinates were used as explanatory variables in linear mixed-effects models to investigate the relationship between the activity behaviour composition and each cardio-metabolic risk factor. Intra class correlation coefficients of > 0.05 indicated that the data was clustered by country and ethnicity. Subsequently, linear mixed models were used to account for the effect of country and ethnicity on the outcome variables. Models were fitted using restricted maximum likelihood methods using the R package lme4 [31]. Likelihood ratio tests were used to determine the significance of random effects within the model while significant *p*-values for fixed effects were derived using Satterthwait approximations for degrees of freedom [32]. Sociodemographic variables, age, sex, ethnicity, smoking status, intervention centre, education level, household income and status of antihypertensive and lipid lowering medications were included in the model as covariates (all covariates described in Supplementary Material). Intervention site and ethnicity were included as random effects. The dependant variables were HOMA-IR, insulin, FPG, 2 h glucose, HbA_1c_, WC, triglycerides, total cholesterol, HDL-C, LDL-C and hs-CRP.

The isometric log-ratio multiple linear mixed models were used to predict cardio-metabolic health measures for the mean daily movement behaviour composition. Predicted values of each cardio-metabolic marker were then calculated for new compositions where fixed durations of time had been reallocated from one movement behaviour to another while the remaining behaviours were kept constant [18]. The new predicted values were then subtracted from the mean composition to find the difference in cardio-metabolic marker after the reallocation of time between behaviours [18]. Furthermore, a sensitivity analyses was performed to assess whether associations with cardio-metabolic markers differed between long and short sleepers using a median split.

All regression models were checked for linearity, normality, homoscedasticity and outlying observations to ensure assumptions were not violated. Due to their positively skewed distribution, triglycerides, insulin and HOMA-IR were square root transformed while hs-CRP was logarithmically transformed (log_10_).

Two-sample Kolmogorov-Smirnov tests were performed between the predicted value of the dependant variable before and after the reallocation of time between behaviours. Therefore, repeated tests following the sequential reallocation of 1 min between behaviours indicated how much time needed to be reallocated before a significant difference was detected.

## Results

Of the 2326 eligible participants, 1699 met the accelerometer wear time criteria, 237 had acute inflammation (hs-CRP > 10 mg**·**l^− 1^) and were excluded. Finally, 1462 were included in the analysis. Included participants were significantly older (*p* < 0.001), had lower BMI (p < 0.001), WC (*p* = 0.017) and body fat % (*p* = 0.005) than the excluded participants. Furthermore, a greater proportion of men achieved sufficient wear time than women (79.4 and 74.4% respectively, *p* = 0.009). Mean age was 52.3y and 66% were female. Mean BMI, insulin, glucose and HbA_1c_ were 34.9 kg·m^2^, 13.3 ± 7.8 mU·l^− 1^, 6.2 ± 0.7 mmol**·**l^− 1^ and 5.5 ± 0.4% respectively. Descriptive characteristics are presented in full in Supplementary Table 1.

The proportion of time spent in each behaviour derived through standards and compositional statistics are presented in Table 1. The compositional mean represents each behaviour as a relative proportion of the whole. The compositional mean adjusted to 24 h provides the mean value of the composition in minutes, maintaining the ratio between parts.

**Table 1:** Arithmetic and compositional mean of movement behaviours from 1699 adults with pre-diabetes

	Sleep	ST	LIPA	MVPA
Arithmetic mean (SD) minutes	475.5 (73.2)	586.6 (87.0)	310.4(81.1)	28.7 (20.4)
Compositional mean	0.343	0.423	0.219	0.016
Compositional mean minutes adjusted to 24 h	493.3	609.3	314.9	22.5

*ST* sedentary time; *LIPA* light intensity physical activity; *MVPA* moderate-to-vigorous physical activity

The variation of all pairwise log ratios displays the relative dispersion structure (Supplementary Table 2). The highest log-ratio variances all include MVPA which shows that MVPA is the least dependent on the other behaviours. The small log-ratio variances between sedentary time, sleep and LIPA indicate more consistent proportionality (co-dependency) between these behaviours.

The combined effect of the movement behaviours was significantly associated with BMI, body fat %, WC, triglycerides, insulin, HOMA-IR and hs-CRP (Table 2). Compositional isotemporal substitution was carried out for all outcome variables that were associated with the activity composition at an alpha of 0.05. Table 3 shows the predicted change in cardio-metabolic risk markers around its mean, following a 10-min reallocation of time from the behaviour in the column to the behaviour in the row while holding other behaviours constant. The greatest predicted increase in markers of obesity (BMI, WC and body fat %), were observed when MVPA was replaced by ST, however, the magnitude of change when MVPA was replaced by ST, sleep or LIPA were comparable. Similarly, the greatest predicted reductions in all markers of obesity were observed when MVPA was increased at the expense of ST, though, increasing MVPA predicted favourable levels of risk markers regardless of which behaviour was displaced. Reallocating 10 min from any behaviour to ST was associated with an increase in all obesity markers with the greatest predicted change observed when ST replaced MVPA. Reallocating 10 min to sleep predicted a small reduction in obesity markers when displacing ST but not when displacing LIPA or MVPA. A small reduction in predicted level of all markers of obesity was also apparent when LIPA displaced ST but not when LIPA displaced sleep.

**Table 2:** Analysis of variance for the contribution of the 24 h time use composition to the explanation of variance in each cardio-metabolic risk factor

Dependant variable	Sum sq.	df	Den df	F-value	*p*-value
BMI	689.65	3	1243.6	13.17	**< 0.001**
Waist	6052.80	3	1260.5	18.20	**< 0.001**
Body fat %	1514.1	3	1255.2	20.23	**< 0.001**
Triglycerides ^sqrt^	0.35	3	1258.1	2.63	**0.048**
Glucose fasting	0.35	3	1302.1	0.39	0.757
Glucose 2 h	18.442	3	1285.8	1.63	0.181
Insulin ^sqrt^	9.115	3	1247.8	6.55	**< 0.001**
HOMA-IR ^sqrt^	2.48	3	1222.3	5.50	**< 0.001**
HDL-C	0.29	3	1268.7	1.75	0.155
LDL-C	5.03	3	1252.9	2.28	0.085
Total cholesterol	5.21	3	1271.7	2.23	0.083
hs-CRP ^**Log**10^	1.21	3	1252.4	3.95	**0.008**
HbA1c	0.13	3	1245.3	0.57	0.636
Systolic BP	220.4	3	1276.4	0.41	0.746
Diastolic BP	184.26	3	1264.7	0.84	0.470

All models were adjusted for: age, sex, income, education, medication status ethnicity* and site *. All models that did not contain BMI or fat% as the Dv were additionally adjusted for BMI. * = random effects

*BMI* body mass index, *BP* blood pressure, *HbA*_*1c*_ haemoglobin A1c, *HOMA-IR* homeostatic model assessment of insulin resistance, *HDL-C* high density lipoprotein cholesterol, *LDL-C* low density lipoprotein cholesterol, *CRP* C-reactive protein, *Den df* Denominator degrees of freedom

**Table 3:** Predicted change in each outcome following the reallocation of 10-min from the behaviour in the column to the behaviour in the row

	Sleep	95% CI		ST	95% CI		LIPA	95% CI		MVPA	95% CI	
BMI												
Sleep				−0.16*	(− 0.26 to − 0.06)		−0.05	(− 0.16 to 0.06)		1.17*	(0.67 to 1.68)	
ST	0.16*	(0.05 to 0.26)					0.11*	(0.01 to 0.21)		1.13*	(0.87 to 1.82)	
LIPA	0.05	(−0.06 to 0.16)		−0.11*	(− 0.20 to − 0.01)					1.22*	(0.70 to 1.74)	
MVPA	−0.73*	(−1.06 to − 0.4)		−0.89*	(− 1.21 to − 0.57)		−0.78*	(− 1.13 to − 0.43)				
Waist												
Sleep				−0.10*	(− 0.18 to − 0.02)		−0.02	(− 0.1 to 0.07)		1.28*	(0.88 to 1.68)	
ST	0.10*	(0.02 to 0.18)					0.08*	(0.01 to 0.16)		1.38*	(0.99 to 1.77)	
LIPA	0.02	(−0.07 to 0.10)		−0.09*	(− 0.16 to − 0.01)					1.29*	(0.88 to 1.71)	
MVPA	−0.81*	(−1.07 to − 0.55)		−0.91*	(− 1.16 to − 0.66)		−0.82*	(− 1.1 to − 0.55)				
Fat%												
Sleep				−0.08	(− 0.17 to 0.01)		0.06	(− 0.03 to 0.16)		1.30*	(0.88 to 1.71)	
ST	0.08	(−0.01 to 0.17)					0.14*	(0.06 to 0.23)		1.38*	(0.97 to 1.78)	
LIPA	−0.06	(−0.15 to 0.03)		−0.14*	(− 0.22 to − 0.06)					1.23*	(0.81 to 1.66)	
MVPA	−0.83*	(−1.1 to − 0.56)		−0.91*	(−1.17 to − 0.65)		−0.76*	(− 1.05 to − 0.48)				
^sqrt^ Insulin												
Sleep				0.02	(−0.16 to 0.19)		0.29*	(0.10 to 0.48)		0.96*	(0.1 to 1.83)	
ST	−0.02	(− 0.2 to 0.16)					0.28*	(0.11 to 0.44)		0.95*	(0.1 to 1.79)	
LIPA	−0.29*	(− 0.48 to − 0.1)		−0.27*	(− 0.44 to − 0.1)					0.68	(−0.22 to 1.57)	
MVPA	−0.65*	(−1.21 to − 0.09)		−0.63*	(− 1.18 to − 0.08)		−0.35	(− 0.95 to 0.24)				
^sqrt^ HOMA-IR												
Sleep				0.04	(−0.16 to 0.23)		0.28*	(0.07 to 0.49)		0.78	(−0.17 to 1.73)	
ST	0.04	(−0.16 to 0.23)					0.32*	(0.14 to 0.51)		0.82	(−0.11 to 1.76)	
LIPA	−0.28*	(−0.48 to − 0.07)		−0.32*	(− 0.5 to − 0.13)					0.51	(−0.47 to 1.49)	
MVPA	−0.52	(−1.14 to 0.1)		−0.56	(− 1.16 to 0.04)		− 0.24	(− 0.89 to 0.41)				
^sqrt^ Triglycerides												
Sleep				−0.06	(− 0.22 to 0.090)		0.01	(− 0.16 to 0.170)		0.90*	(0.15 to 1.650)	
ST	0.06	(−0.1 to 0.220)					0.07	(−0.08 to 0.220)		0.97*	(0.23 to 1.70)	
LIPA	0.01	(−0.17 to 0.160)		−0.07	(− 0.22 to 0.080)					0.90*	(0.12 to 1.670)	
MVPA	−0.57*	(−1.06 to − 0.08)		−0.64*	(−1.11 to − 0.170)		−0.57*	(− 1.08 to − 0.050)				
^Log10^ hs-CRP												
Sleep				−0.17	(− 0.75 to 0.41)		0.25	(− 0.37 to 0.86)		3.93*	(1.19 to 6.68)	
ST	0.17	(−0.42 to 0.75)					0.41	(−0.13 to 0.96)		4.10*	(1.42 to 6.78)	
LIPA	−0.24	(−0.85 to 0.37)		− 0.41	(− 0.95 to 0.13)					3.69*	(0.85 to 6.53)	
MVPA	−2.53*	(−4.33 to −0.74)		− 2.7*	(− 4.43 to − 0.98)		−2.28*	(−4.18 to − 0.39)				

Values represent % change around the mean

*ST* sedentary time, *LIPA* light intensity physical activity, *MVPA* moderate-to-vigorous physical activity, *BMI* body mass index, *HOMA-IR* homeostasis model assessment for insulin resistance, *hs-CRP* high sensitivity C-reactive protein, * indicates statistical significant change in risk marker

Light intensity activity was beneficially associated with insulin and HOMA-IR, when replacing ST and sleep but not MVPA. For all outcome variables, the magnitude of predicted improvements was greater when sleep and ST were replaced by MVPA.

Although sleep was not associated with any markers of cardio-metabolic health, the predicted detriment of reducing MVPA and LIPA on triglycerides, hs-CRP and markers of obesity were reduced if sleep was increased instead of ST. Indeed, sleep was beneficially associated with BMI and WC but only when replacing ST.

Sensitivity analysis revealed that the patterns of association differed between long and short sleepers (Supplementary Tables 4, 5, 6 and 7). For long sleepers, the combined effect of the movement behaviours was significantly associated with BMI, body fat %, WC, triglycerides, insulin and HOMA-IR while for short sleepers it was only associated with BMI, body fat %, and WC.

Compositional isotemporal substitution revealed that for short sleepers, replacing ST with sleep predicted favourable levels of BMI. Conversely, replacing sleep with LIPA predicted favourable BMI levels for long sleepers but not short sleepers. Similarly, for long sleepers but not short sleepers replacing sleep or ST with LIPA predicted beneficial levels of HOMA-IR (Supplementary Tables 6 and 7).

**Fig. 1:**
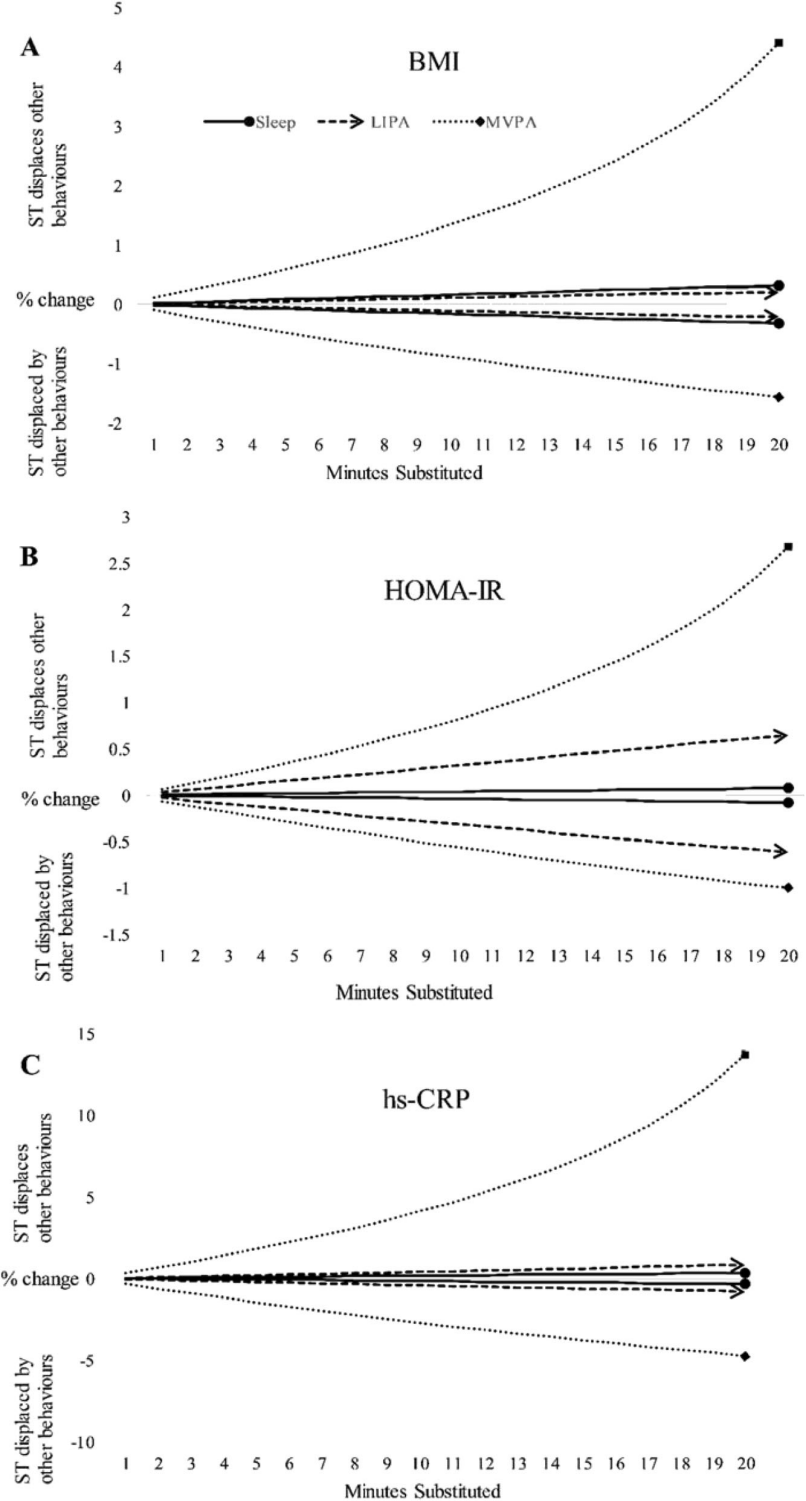
Asymmetry of predicted change in outcome variables with the reallocation of time to and from sedentary time. Figure **1-a** shows the predicted change in BMI, with the reallocation of time to/from ST. As MVPA increases at the expense of ST, predicted BMI steadily declines. Conversely, as MVPA is displaced by ST, predicted BMI rises exponentially. Figures **1-a** shows that relative to MVPA, displacing ST with sleep or LIPA is associated with only marginal change in BMI. However, figure **1-b** suggests that displacing ST with LIPA represents a favourable alternative to ST or sleep. ST sedentary time, LIPA light intensity physical activity, MVPA moderate-to-vigorous physical activity, BMI body mass index, HOMA-IR homeostasis model assessment for insulin resistance, hs-CRP, high sensitivity C-reactive protein

The predicted change in risk markers following the reallocation of time from one behaviour to another was non-symmetrical. For example, reallocating 10 min from ST to MVPA was associated with 2.5% reduction in hs-CRP. Conversely, replacing 10 min of MVPA with ST was associated with a 4.1% increase in hs-CRP. This asymmetry was consistent for all metabolic outcomes and is clearly presented in Fig. 1.

Results from the Kolmogorov-Smirnov tests (Table 4) showed that the reallocation of 4-min from ST to MVPA produced a significant difference in the distribution of predicted BMI and WC, while 8-min was required to significantly reduce body fat %. Except for insulin and HOMA-IR, all markers of cardio-metabolic health that were significantly associated with the daily time use composition were significantly changed with the reallocation of < 10 min to/from MVPA and ST. When LIPA was increased at the expense of ST, a greater reallocation of time was required to produce a significant difference in the distribution of all outcome variables (Supplementary Table 3). The reallocation of 35 min led to a significant difference in HOMA-IR, ≥45 min for BMI while ≥70 min was needed to significantly reduce predicted triglycerides and hs-CRP.

**Table 4:** Comparison of the sample distribution of each DV and predicted values following the reallocation of time from ST to MVPA

Minutes reallocated	BMI		Waist		Body fat		Insulin		HOMA-IR		2 h Glucose		Triglycerides		HDL-C		hs-CRP	
	*D*	*P*	*D*	*P*	*D*	*P*	*D*	*P*	*D*	*P*	*D*	*P*	*D*	*P*	*D*	*P*	*D*	*P*
1	0.021	0.871	0.022	0.847	0.013	0.995	0.009	1	0.008	1	0.011	1	0.017	0.981	0.008	1	0.012	0.999
2	0.034	0.343	0.031	0.443	0.023	0.821	0.013	0.995	0.012	0.998	0.016	0.987	0.027	0.615	0.011	1	0.018	0.961
3	0.044	0.108	0.044	0.099	0.027	0.646	0.017	0.981	0.017	0.972	0.021	0.871	0.037	0.241	0.012	0.999	0.024	0.765
4	**0.056**	**0.016**	**0.054**	**0.023**	0.032	0.416	0.020	0.914	0.021	0.893	0.027	0.645	0.047	0.063	0.014	0.998	0.028	0.585
5	**0.065**	**0.003**	**0.064**	**0.004**	0.038	0.224	0.023	0.793	0.024	0.765	0.029	0.526	**0.053**	**0.025**	0.017	0.981	0.034	0.321
6	**0.076**	**0.001**	**0.073**	**0.001**	0.042	0.140	0.025	0.706	0.027	0.615	0.033	0.366	**0.061**	**0.006**	0.018	0.961	0.040	0.178
7					0.047	0.063	0.028	0.585	0.030	0.497	0.036	0.260	**0.068**	**0.002**	0.018	0.961	0.042	0.128
8					**0.052**	**0.031**	0.029	0.526	0.032	0.416	0.039	0.192			0.020	0.914	0.047	0.063
9					**0.055**	**0.018**	0.032	0.416	0.034	0.343	0.042	0.128			0.021	0.871	**0.052**	**0.031**
10					**0.059**	**0.009**	0.034	0.343	0.036	0.279	0.046	0.083			0.023	0.793	**0.055**	**0.018**
11							0.035	0.299	0.037	0.241	0.048	0.057			0.026	0.676	**0.059**	**0.009**
12							0.037	0.241	0.040	0.178	**0.052**	**0.031**			0.029	0.555		
13							0.038	0.208	0.041	0.151	**0.055**	**0.018**			0.031	0.442		
14							0.040	0.164	0.043	0.118	**0.058**	**0.010**			0.032	0.416		
15							0.042	0.139	0.046	0.083					0.034	0.343		
16							0.044	0.099	0.048	0.057					0.034	0.321		
17							0.046	0.083	0.050	0.052					0.035	0.299		
18							0.047	0.063	**0.053**	**0.025**					0.036	0.279		
19							0.049	0.052	**0.055**	**0.018**					0.037	0.241		
20							**0.049**	**0.047**							0.038	0.208		

Two sample Kolmogorov-Smirnov test comparing the sample distribution between predicted value of each dependant variable and the predicted value with the sequential reallocation of 1 min from ST to MVPA

*ST* sedentary time, *MVPA* moderate-to-vigorous physical activity, *BMI* body mass index, *HOMA-IR* homeostasis model assessment for insulin resistance, *HDL-C* high density lipoprotein cholesterol, *hs-CRP* high sensitivity C-reactive protein

## Discussion

This study showed that in overweight and obese adults with pre-diabetes the daily time use composition of sleep, ST, LIPA and MVPA was significantly associated with BMI, body fat %, WC, triglycerides, insulin, HOMA-IR and hs-CRP. Compositional isotemporal substitution models consistently showed MVPA to be the most important behaviour within the composition that was beneficially associated with cardio-metabolic health markers. The greatest predicted improvements were observed when MVPA was increased by 10 min at the expense of ST. Indeed, when the data were modelled, reducing ST or increasing MVPA was associated with favourable change in cardio-metabolic health markers irrespective of the behaviour being exchanged. These findings are consistent with similar studies using compositional isotemporal substitution in healthy adults [11], older adults [33] older women [17] and adolescents [16] and supports public health guidelines that recommend spending time in MVPA and minimizing prolonged sitting [34]. Our results also demonstrated that replacing 10-min of ST with LIPA, although less pronounced than MVPA, predicted significant differences in BMI, WC, body fat%, insulin and HOMA-IR. Furthermore, the magnitude of difference when replacing MVPA with ST was greater than with LIPA suggesting that LIPA may provide some health benefits. LIPA contributes substantially to daily energy expenditure [35] and in combination with sedentary time occupied the majority of waking hours. Increasing LIPA may be a pragmatic approach to breaking up ST and improving cardio-metabolic health in populations who have low levels MVPA.

Despite the statistically significant change in predicted risk markers after substituting 10-min from ST to LIPA or sleep, the magnitude of this change was minimal when BMI, body fat%, triglycerides and hs-CRP are the outcome variables and there is little difference between sleep and LIPA (Fig. 1). However, for HOMA-IR replacing ST with LIPA offers a significant improvement compared to sleep, suggesting that LIPA may offer benefits for glycaemic control.

In a recent review, LIPA was shown to be favourably associated with WC, triglyceride, insulin and the presence of metabolic syndrome after controlling for MVPA [36]. However, the studies did not use CoDa. Therefore, the co-dependence of time-use domains was not accounted for and the effects of LIPA were not independent of the confounding effects of sleep and ST. The few studies that have used CoDA to assess the combined effects of time spent in sleep, ST, LIPA and MVPA on health markers show inconsistent results. In a study of older adults, Pelclová and colleagues [17] reported that the time use composition was significantly associated with obesity markers. However, reallocating 30 min of ST to LIPA did not significantly reduce body fat percent or BMI. Similarly, in a study of older Australian adults, the reallocation of time from ST to LIPA showed no significant associations with BMI or waist to hip ratio [33]. The lack of effect found in these studies may have been due to their small sample size resulting in the lack of statistical power. Furthermore, neither of these studies tested diabetes risk variables such as fasting insulin or HOMA-IR. In contrast, Chastin and colleagues [11] found that LIPA was favourably associated with LDL-C, triglycerides, fasting glucose, insulin and HOMA, which became more pronounced when LIPA replaced ST as opposed to sleep. Moreover, interventions have shown that replacing ST with light ambulatory activity or postural changes such as standing can improve glycaemic control to a greater extent than structured exercise of the same energy cost albeit not in those with diagnosed pre-diabetes [37]. In a randomised control trial of people who were overweight/obese and sedentary, Houmard et al. [38] showed that physical activity improved insulin sensitivity at all intensities and volumes compared to controls. However, exercise duration had the greatest effect on insulin sensitivity regardless of intensity or volume. Similarly, Duvivier and colleagues [39] demonstrated that in sedentary subjects, minimal intensity physical activity such as standing or light walking maintained for a longer duration were associated with improved insulin sensitivity and plasma lipids to greater extent than shorter periods of MVPA of comparable energy cost. Thus, it appears that replacing ST with LIPA could offer a pragmatic approach to promote glycaemic control in adults with pre-diabetes and the results of this study confirm these findings.

The observed estimates for the reallocation of time around the average composition were asymmetrical. Previous studies using compositional isotemporal substitution in healthy adults [11], and those at risk of type 2 diabetes [19] have reported asymmetrical estimates. This has in part been attributed to the relative contribution of each activity behaviour to the daily composition (24 h). For example, a 10-min reallocation represents a substantially larger relative change in MVPA than it does in ST or sleep [4, 11, 18]. This asymmetry demonstrates that reducing activity levels below the mean had a greater predicted detriment to cardiometabolic risk than the predicted benefit following an equivalent increase above the mean.

These findings suggest that besides the promotion of MVPA, maintaining existing levels of MVPA is of great importance. This may be particularly pertinent in those with pre-diabetes given the progressive nature of type 2 diabetes particularly in adults above the age of 40 years, when activity levels typically decline [23].

The different patterns of association observed between long and short sleepers suggests a greater influence of the LIPA and MVPA on BMI and HOMA-IR among long sleepers. Similar findings were reported by Biddle et al. [19] who found a significant association between stepping and insulin sensitivity in long but not short sleepers. Further research is required to assess the patterns of association between sleep time and cardiometabolic health in the context of the daily time use composition.

Due to the large sample size and narrow confidence intervals, small reallocations of time that produced relatively modest magnitudes of change in the outcome variable were deemed significant. To prevent over reliance on these confidence intervals to infer significance, Kolmogorov-Smirnov tests were used to estimate how much time needed to be reallocated before the distributions were significantly different. The reallocation of 4-min from ST to MVPA produced a significant difference in the distribution of predicted BMI and WC, while 8-min was required to significantly reduce body fat %. Except for insulin and HOMA-IR, all markers of cardio-metabolic health that were significantly associated with the daily time use composition were significantly changed with the reallocation of < 10 min to/from MVPA and ST. PA guidelines recommend accumulating ≥150 min MVPA per week in bouts lasting ≥10miutes [40]. However, several studies demonstrating a reduced risk of all-cause-mortality related to total PA volume accumulated irrespective of bout length [41, 42] have lead the requisite for ≥10 min bouts being retracted [43]. In agreement with this premise, the current analysis demonstrated that the reallocation of between 4 and 9 min from ST to MVPA produced a significant change in the distribution of BMI, WC, body fat%, triglycerides, LDL-C and hs-CRP. However, to produce a significant change in insulin and HOMA-IR it was predicted that the reallocation of ≥19 min was required (Supplementary Table 3). This concurs with intervention studies which have shown duration to be an important factor in improving insulin sensitivity [37, 38]. When LIPA was increased at the expense of ST, a greater reallocation of time was required to produce a significant difference in the distribution of all outcome variables (Supplementary Table 3S). Interestingly, the minimum time reallocation required to produce a significant difference was observed for diabetes risk markers. For example, replacing 35-min of ST with LIPA produced a significant change in predicted HOMA-IR.

Strengths of this study include the large multi-national sample using standardised measurements across 8-study sites. The inclusion of a 24-h accelerometer wear time protocol providing objective estimates of each component of the time use composition. Furthermore, we used CoDa which accounts for the collinear nature of compositional data. To our knowledge, this is the first study to use CoDa to explore associations between 24-h time use data and cardio-metabolic risk factors in adults with pre-diabetes.

The study also has several limitations, while accelerometers offer more robust assessments of physical activity than self-report [44], hip worn accelerometers may not detect some types of activity such as cycling, or water-based activities when the device is removed. Furthermore, they do not distinguish between postural changes such as lying, sitting and standing still. In the current study Troiano cut-points were used [23], there are no widely accepted accelerometer cut-points for adults with overweight or obesity. Given that the metabolic cost of walking increases with body weight, the relative exercise intensity in this cohort is likely to be higher compared to a healthy population. Consequently, it is possible that MVPA may have been underestimated due to these cut-points being determined in adults of healthy weight.

It is also important to emphasize that the cross-sectional design does not allow insight into the direction of causality. Reducing physical activity and increasing sedentary time may lead to elevations in cardiometabolic risk factors. However, the reverse is also plausible. For example, being overweight/obese may make physical activity more difficult. Therefore, studies of high-risk populations are particularly susceptible to reverse causality.

Predictions from the linear models do not represent change in cardio-metabolic risk markers following isotemporal changes in the time use composition. Instead, levels of cardio-metabolic risk markers are predicted for a given daily time use composition. Data lost due to non-compliance with the accelerometer wear time protocol was relatively high (see Supplementary Material), this selection bias may limit the generalizability of our findings.

Finally, although analyses were controlled for socio-demographic confounders there might have been some residual confounding from unmeasured variables such as dietary factors.

## Conclusions

In an international sample of adults with pre-diabetes, the daily composition of sleep, ST, LPA, and MVPA were collectively associated with diabetes risk. Our analysis found that replacing MVPA with any other behaviour around the mean movement composition predicted a greater cardio-metabolic risk. Conversely, increasing MVPA at the expense of sleep, ST or LPA, predicted beneficial levels cardio-metabolic risk but the magnitude of the differences was smaller. Further replacing ST with LIPA was also associated with beneficial levels of cardio-metabolic risk markers, most notably insulin and HOMA-IR. Sleep was beneficially associated with all markers of obesity and hs-CRP but only when replacing ST. These findings provide further evidence for the role of MVPA in the prevention of type 2 diabetes but also suggest the public health message should emphasize the importance of maintaining existing levels of MVPA (ie keep moving, not necessarily moving more). Our analysis also suggests that, replacing ST with LIPA was associated with beneficial levels of cardio-metabolic risk markers, most notably insulin and HOMA-IR.

These findings have important clinical and public health implications, as they indicate that replacing ST with LIPA may produce metabolic benefits that could contribute to the prevention and management of type 2 diabetes. Increasing LIPA may also be more achievable in individuals who are obese or overweight than increasing MVPA. While MVPA confers greater health benefits, for individuals who are unable or unwilling to engage in MVPA, increasing LIPA may represent a pragmatic way to improve diabetes risk.

## Supplementary information

**Additional file 1.** Data flow chart and covariates.

**Additional file 2.** Participant characteristics.

**Additional file 3.** Variation matrix.

**Additional file 4.** Comparison of the sample distribution of each DV and predicted values following the reallocation of time from ST to LIPA.

**Additional file 5.** Asymmetry of predicted change in outcome variables.

**Additional file 6.** Sensitivity analysis of long and short sleepers.

## Data Availability

The datasets generated during and/or analysed during the current study are not publicly available but are obtainable from the corresponding author on reasonable request.

## Abbreviations

BMI: Body mass Index
CoDa: Compositional Data Analysis
FPG: Fasting Plasma Glucose
HDL-C: High Density Lipoprotein Cholesterol
HOMA-IR: Homeostasis Model Assessment for Insulin Resistance
hs-CRP: High Sensitivity C-reactive Protein
IFG: Impaired Fating Glucose
IGT: Impaired glucose tolerance
LIPA: Light Intensity Physical Activity
MVPA: Moderate to Vigorous Physical Activity
OGTT: Oral Glucose Tolerance Test
PREVIEW: PREVention of diabetes through lifestyle Intervention and population studies in Europe and around the World
ST: Sedentary Time
WC: Waist Circumference
