# Supplementary material for: Compositional analysis of the associations between 24-h movement behaviours and cardio-metabolic risk factors in overweight and obese adults with pre-diabetes from the PREVIEW study: cross-sectional baseline analysis
Source: Int J Behav Nutr Phys Act. 2020 Mar 4;17:29. doi: 10.1186/s12966-020-00936-5 (PMC7055067; doi:10.1186/s12966-020-00936-5)
Supplement: Supplementary file 1 — Additional file 1. Data flow chart and covariates. [file 12966_2020_936_MOESM1_ESM.docx]

Online-Only Supplemental Material

Valid accelerometer data with wear time and sleep data available for 1,699 individuals

PREVIEW participants eligible at baseline 2,326

2,083 wore accelerometers

1,924 with sufficient wear time

Excluded 159 invalid accelerometery or insufficient wear time

243 no accelerometer data

Excluded 225 missing sleep data

Data flow chart

1,462 participants included in the final analysis

Excluded 237 hs-CRP >10 **mg·l^-1^**

**Measurements of body composition at the intervention sites**:

- Copenhagen, iDXA, software v. 15, GE-Lunar, Madison, WI;
- Helsinki, BIA InBody720 Body Composition Analyzer by Biospace Co., Ltd, manufacturing year 2004;
- Nottingham, DXA GE Lunar prodigy with eCORE 2005 software (version 9.30.044);
- Maastricht, BodPod: Life Measurement Corporation, Inc, Concord, CA, USA;
- Navarra, Lunar iDXA Software: EnCORE v. 14.10.022 (in a subsample 59), the remaining Spanish participants were measured with a TANITA BC-420MA (TANITA corp);
- Sophia, BIA, TANITA BC-420MA (TANITA corp.);
- Auckland, iDXA, software v. 15, GE-Lunar, Madison, WI;
- Sydney, DXA, Hologic Discovery W, Bedford, MA, USA with Hologic APEX Software (version 4, Hologic Inc.).

The measurements were completed according to the manufacturer’s instructions.

**Covariates included in Mixed models**

- Age: continuous (years)
- Sex: Male, Female
- Ethnicity: Caucasian, Asian, Black, Arabic, Hispanic, other
- Smoking status: yes (daily), sometimes (less than daily), no
- Intervention centre: Copenhagen, Helsinki, Maastricht, Nottingham, Navarra, Sophia, Sydney, Australia),
- Education level: No formal education, primary or junior school, secondary school, secondary vocational education, higher vocational education, university education
- Household income: Less than €13,200, €13,200–€17,000, €17,001–€20,500, €20,501–€24,200, €24,201–€28,600, €28,601–€33,500, €33,501–€39,100, €39,101–€46,400, €46,401–€52,800, €52,801 or more per year).
