# Supplementary material for: Compositional analysis of the associations between 24-h movement behaviours and cardio-metabolic risk factors in overweight and obese adults with pre-diabetes from the PREVIEW study: cross-sectional baseline analysis
Source: Int J Behav Nutr Phys Act. 2020 Mar 4;17:29. doi: 10.1186/s12966-020-00936-5 (PMC7055067; doi:10.1186/s12966-020-00936-5)
Supplement: Supplementary file 2 — Additional file 2. Participant characteristics. [file 12966_2020_936_MOESM2_ESM.docx]

| **Table 1S** | | **Metabolic and physical activity characteristics of 1,462 pre-diabetic adults** | | | |
| --- | --- | --- | --- | --- | --- |
| ***Characteristics*** | | | ***Mean*** |  | ***SD*** |
| Age (years) | | | 52.8 | ± | 11.1 |
| BMI (kg·m^2^) | | | 34.5 | ± | 5.4 |
| Body fat (%) | | | 43.1 | ± | 7.5 |
| Waist circumference (cm) | | | 108.9 | ± | 15.2 |
| Systolic BP (mmHg) | | | 129.2 | ± | 15.7 |
| Diastolic BP (mmHg) | | | 78.1 | ± | 10.8 |
| Fasting insulin (mU·l^-1^) | | | 13.0 | ± | 7.6 |
| Fasting plasma glucose (mmol**·**l^-1^) | | | 6.2 | ± | 0.7 |
| 2-h plasma glucose (mmol**·**l^-1^) | | | 7.6 | ± | 2.2 |
| HbA_1c_ (mmol**/**mol) | | | 36.8 | ± | 3.9 |
| HbA_1c_ (%) | | | 5.5 | ± | 0.3 |
| HOMA-IR | | | 3.6 | ± | 2.3 |
| Triglycerides (mmol**·**l^-1^) | | | 1.5 | ± | 0.8 |
| Total cholesterol (mmol**·**l^-1^) | | | 5.2 | ± | 1.0 |
| HDL-cholesterol (mmol**·**l^-1^) | | | 1.3 | ± | 0.3 |
| LDL-cholesterol (mmol**·**l^-1^) | | | 3.2 | ± | 0.9 |
| Hs-CRP (mg**·**l^-1^) | | | 3.3 | ± | 2.3 |
| **Accelerometer variables** | | |  |  |  |
| *Waking wear Time* *(minutes·day^-1^)* | | | 927.4 | ± | 72.9 |
| *Sleep (minutes·day^-1^)* | | | 474.1 | ± | 72.7 |
| *Sedentary (minutes·day^-1^)* | | | 584.2 | ± | 86.7 |
| *Light (minutes·day^-1^)* | | | 312.6 | ± | 80.5 |
| *MVPA (minutes·day^-1^)* | | | 29.7 | ± | 20.6 |
| **Sex** | | ***%*** | | |  |
| *Female* | | 64.6 | | |  |
| *Male* | | 35.4 | | |  |
| **Ethnicity** | |  | | |  |
| *Caucasian* | | | 89.7 | | |
| *Asian* | | | 2.5 | | |
| *Black* | | | 1.9 | | |
| *Arabic* | | | 0.3 | | |
| *Hispanic* | | | 2.0 | | |
| *Other* | | | 3.7 | | |
| **Smoking** | | |  | | |
| *Yes (Daily)* | | | 8.1 | | |
| *Sometimes (less than weekly)* | | | 3.3 | | |
| *No* | | | 88.6 | | |
| **Education** | | |  | | |
| *No formal education* | | | 0.2 | | |
| *Primary/junior school* | | | 2.3 | | |
| *Secondary school* | | | 14.5 | | |
| *Secondary vocational education* | | | 17.7 | | |
| *Higher vocational education* | | | 19.0 | | |
| *University education* | | | 36.8 | | |
| *Other* | | | 9.4 | | |
| **Medication** | | |  | | |
| Using antihypertensive medication | | | 21.2 | | |
| Using lipid lowering medication | | | 10.5 | | |
| **Household income (per year)** | | |  | | |
| *less than €13,200* | | | 5.8 | | |
| *€13,200 - €17,000* | | | 4.7 | | |
| €17,001–€20,500 | | | 3.8 | | |
| €20,501–€24,200 | | | 5.0 | | |
| €24,201–€28,600 | | | 5.2 | | |
| €28,601–€33,500 | | | 7.4 | | |
| €33,501–€39,100 | | | 10.0 | | |
| €39,101–€46,400 | | | 11.3 | | |
| €46,401–€52,800 | | | 13.8 | | |
| €52,801 *or more* | | | 32.8 | | |

| BMI, body mass index; BP, blood pressure; HbA_1c_, haemoglobin A1c; HOMA-IR, homeostatic model assessment of insulin resistance; HDL, high density lipoprotein; LDL, low density lipoprotein; hs-CRP, high sensitivity C-reactive protein |
| --- |
