# Supplementary material for: Compositional analysis of the associations between 24-h movement behaviours and cardio-metabolic risk factors in overweight and obese adults with pre-diabetes from the PREVIEW study: cross-sectional baseline analysis
Source: Int J Behav Nutr Phys Act. 2020 Mar 4;17:29. doi: 10.1186/s12966-020-00936-5 (PMC7055067; doi:10.1186/s12966-020-00936-5)
Supplement: Supplementary file 3 — Additional file 3. Variation matrix. [file 12966_2020_936_MOESM3_ESM.docx]

| **Table 2S. Variation matrix of pairwise log-ratio variances** | | | | |
| --- | --- | --- | --- | --- |
|  | Sleep | ST | LIPA | MVPA |
| Sleep | 0.000 | 0.052 | 0.110 | 0.724 |
| ST | 0.052 | 0.000 | 0.129 | 0.762 |
| LIPA | 0.110 | 0.129 | 0.000 | 0.667 |
| MVPA | 0.724 | 0.762 | 0.667 | 0.000 |
| ST, sedentary time; LIPA, light intensity activity; MVPA, moderate to vigorous activity | | | | |

The variation of all pairwise log ratios displays the relative dispersion structure. A value close to zero implies that the two parts in the ratio are highly proportional (co-dependent).
