# Supplementary material for: Compositional analysis of the associations between 24-h movement behaviours and cardio-metabolic risk factors in overweight and obese adults with pre-diabetes from the PREVIEW study: cross-sectional baseline analysis
Source: Int J Behav Nutr Phys Act. 2020 Mar 4;17:29. doi: 10.1186/s12966-020-00936-5 (PMC7055067; doi:10.1186/s12966-020-00936-5)
Supplement: Supplementary file 4 — Additional file 4. Comparison of the sample distribution of each DV and predicted values following the reallocation of time from ST to LIPA. [file 12966_2020_936_MOESM4_ESM.docx]

| Table 3S | **Comparison of the sample distribution of each DV and predicted values following the reallocation of time from ST to LIPA** | | | | | | | | | | | | | | | | | |
| --- | --- | --- | --- | --- | --- | --- | --- | --- | --- | --- | --- | --- | --- | --- | --- | --- | --- | --- |
| Minutes reallocated | BMI | | Waist | | Body fat | | Insulin | | HOMA-IR | | 2h Glucose | | Triglycerides | | HDL-C | | hs-CRP | |
|  | *D* | *P* | *D* | *P* | *D* | *P* | *D* | *D* | *D* | *P* | *D* | *P* | *D* | *P* | *D* | *P* | *D* | *P* |
| 5 | 0.010 | 1 | 0.010 | 1 | 0.007 | 1 | 0.012 | 0.999 | 0.011 | 1 | 0.008 | 1 | 0.007 | 1 | 0.008 | 1 | 0.009 | 1 |
| 10 | 0.014 | 0.998 | 0.015 | 0.995 | 0.012 | 0.999 | 0.019 | 0.947 | 0.019 | 0.947 | 0.011 | 0.999 | 0.013 | 0.999 | 0.012 | 0.999 | 0.012 | 0.999 |
| 15 | 0.019 | 0.948 | 0.019 | 0.932 | 0.016 | 0.987 | 0.025 | 0.736 | 0.027 | 0.645 | 0.016 | 0.992 | 0.017 | 0.981 | 0.016 | 0.992 | 0.016 | 0.987 |
| 20 | 0.026 | 0.676 | 0.024 | 0.765 | 0.019 | 0.948 | 0.030 | 0.497 | 0.034 | 0.321 | 0.019 | 0.958 | 0.020 | 0.932 | 0.019 | 0.947 | 0.019 | 0.932 |
| 25 | 0.032 | 0.391 | 0.027 | 0.615 | 0.021 | 0.871 | 0.036 | 0.279 | 0.040 | 0.178 | 0.022 | 0.847 | 0.023 | 0.821 | 0.022 | 0.847 | 0.023 | 0.793 |
| 30 | 0.037 | 0.242 | 0.032 | 0.391 | 0.025 | 0.706 | 0.040 | 0.164 | 0.047 | 0.069 | 0.024 | 0.736 | 0.026 | 0.676 | 0.025 | 0.736 | 0.026 | 0.676 |
| 35 | 0.042 | 0.140 | 0.037 | 0.242 | 0.028 | 0.585 | 0.045 | 0.090 | **0.053** | **0.025** | 0.028 | 0.5850 | 0.029 | 0.526 | 0.027 | 0.645 | 0.028 | 0.555 |
| 40 | 0.048 | 0.057 | 0.041 | 0.152 | 0.031 | 0.470 | **0.051** | **0.038** | **0.058** | **0.010** | 0.031 | 0.470 | 0.033 | 0.391 | 0.030 | 0.497 | 0.032 | 0.391 |
| 45 | **0.053** | **0.028** | 0.047 | 0.069 | 0.032 | 0.416 | **0.057** | **0.015** | **0.062** | **0.005** | 0.033 | 0.367 | 0.034 | 0.321 | 0.033 | 0.366 | 0.036 | 0.279 |
| 50 | **0.058** | **0.012** | **0.051** | **0.038** | 0.034 | 0.321 | **0.063** | **0.004** |  |  | 0.037 | 0.242 | 0.038 | 0.224 | 0.035 | 0.299 | 0.038 | 0.224 |
| 55 | **0.061** | **0.006** | **0.055** | **0.020** | 0.038 | 0.224 |  |  |  |  | 0.040 | 0.164 | 0.041 | 0.151 | 0.037 | 0.241 | 0.042 | 0.139 |
| 60 |  |  | **0.060** | **0.008** | 0.041 | 0.152 |  |  |  |  | 0.044 | 0.099 | 0.044 | 0.108 | 0.040 | 0.178 | 0.045 | 0.090 |
| 65 |  |  |  |  | 0.043 | 0.118 |  |  |  |  | 0.045 | 0.063 | 0.046 | 0.075 | 0.042 | 0.128 | 0.047 | 0.069 |
| 70 |  |  |  |  | 0.046 | 0.069 |  |  |  |  | **0.049** | **0.048** | **0.050** | **0.042** | 0.046 | 0.083 | **0.051** | **0.035** |
| 75 |  |  |  |  | 0.048 | 0.057 |  |  |  |  | **0.051** | **0.038** | **0.053** | **0.028** | 0.047 | 0.063 | **0.056** | **0.016** |
| 80 |  |  |  |  | **0.051** | **0.035** |  |  |  |  | **0.053** | **0.025** | **0.056** | **0.016** | **0.049** | **0.052** | **0.059** | **0.009** |
| 85 |  |  |  |  | **0.053** | **0.025** |  |  |  |  |  |  |  |  | **0.051** | **0.035** |  |  |
| 90 |  |  |  |  | **0.056** | **0.015** |  |  |  |  |  |  |  |  | **0.055** | **0.018** |  |  |
|  |  |  |  |  |  |  |  |  |  |  |  |  |  |  |  |  |  |  |

| Two sample Kolmogorov-Smirnov test comparing the sample distribution between predicted value of each dependant variable and the predicted value with the sequential reallocation of 5 minutes from ST to LIPA. ST sedentary time, LIPA light intensity physical activity, BMI body mass index, HOMA-IR homeostasis model assessment for insulin resistance, HDL-C high density lipoprotein cholesterol; hs-CRP, high sensitivity C-reactive protein |
| --- |
