# Supplementary material for: Compositional analysis of the associations between 24-h movement behaviours and cardio-metabolic risk factors in overweight and obese adults with pre-diabetes from the PREVIEW study: cross-sectional baseline analysis
Source: Int J Behav Nutr Phys Act. 2020 Mar 4;17:29. doi: 10.1186/s12966-020-00936-5 (PMC7055067; doi:10.1186/s12966-020-00936-5)
Supplement: Supplementary file 5 — Additional file 5. Asymmetry of predicted change in outcome variables. [file 12966_2020_936_MOESM5_ESM.docx]

***
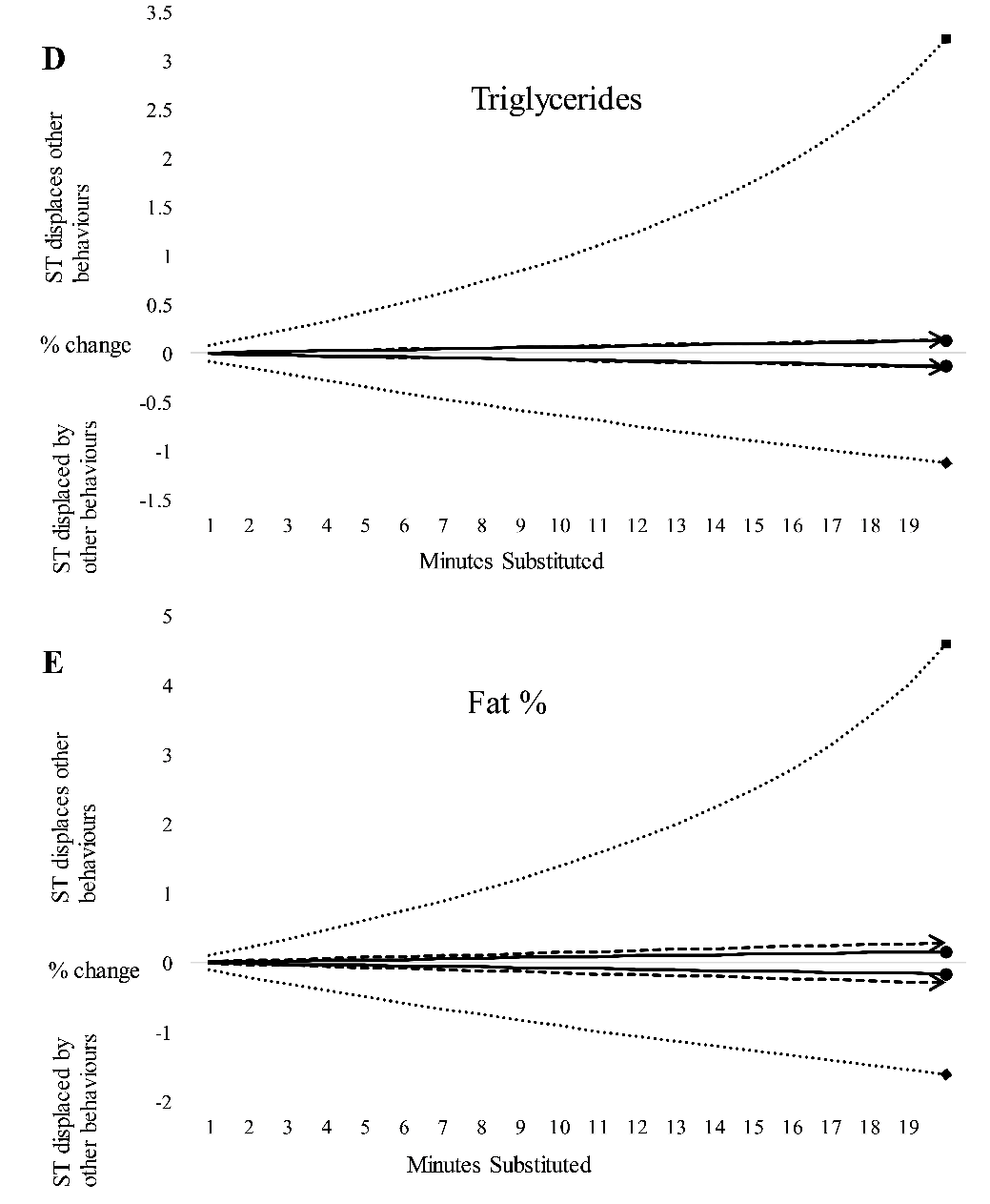
***

Figure 1S. Asymmetry of predicted change in outcome variables with the reallocation of time to and from sedentary time

***
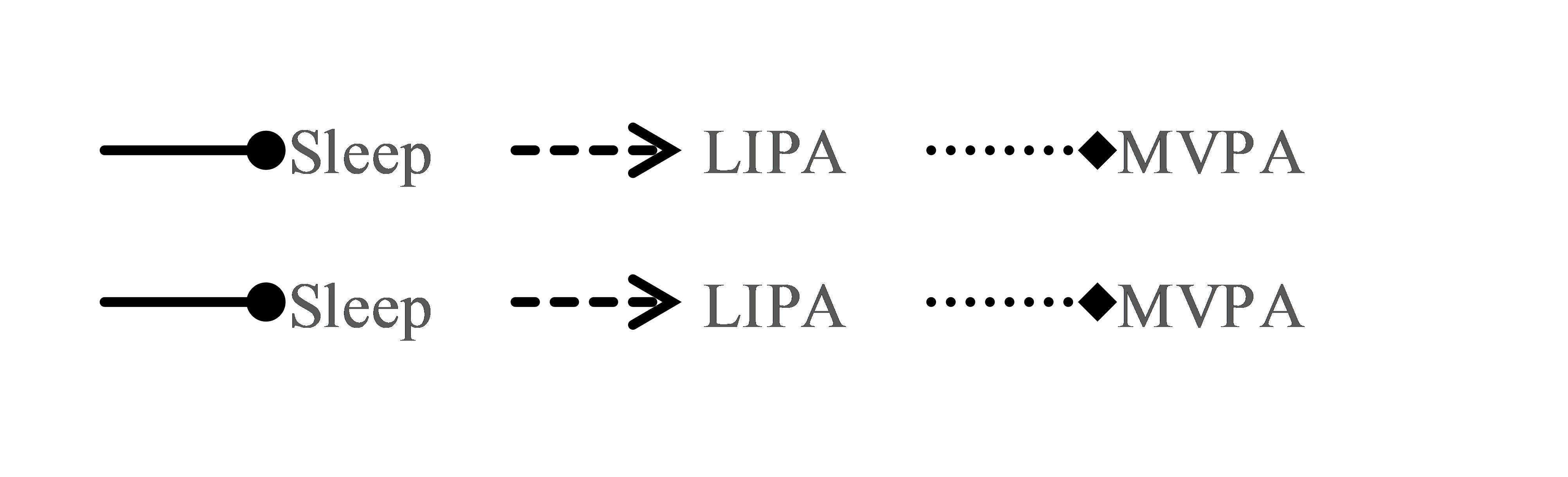
***
