# Supplementary material for: Compositional analysis of the associations between 24-h movement behaviours and cardio-metabolic risk factors in overweight and obese adults with pre-diabetes from the PREVIEW study: cross-sectional baseline analysis
Source: Int J Behav Nutr Phys Act. 2020 Mar 4;17:29. doi: 10.1186/s12966-020-00936-5 (PMC7055067; doi:10.1186/s12966-020-00936-5)
Supplement: Supplementary file 6 — Additional file 6. Sensitivity analysis of long and short sleepers. [file 12966_2020_936_MOESM6_ESM.docx]

| Table 4S | **Analysis of variance for the contribution of the 24h time use composition to the explanation of variance in each cardio-metabolic risk factor for Long sleepers** | | | | | |
| --- | --- | --- | --- | --- | --- | --- |
| **Dependant variable** | | **Sum sq** | **df** | **Den df** | **F-value** | **p-value** |
| BMI | | 437.5 | 3 | 616.3 | 7.38 | **<0.001** |
| Waist circumference | | 2385.5 | 3 | 615.4 | 6.80 | **<0.001** |
| Body fat % | | 646.0 | 3 | 312.3 | 8.09 | **<0.001** |
| Triglycerides ^sqrt^ | | 0.68 | 3 | 484.7 | 5.12 | **0.002** |
| Glucose fasting | | 1.31 | 3 | 625.0 | 1.41 | 0.238 |
| Glucose 2 hour | | 12.3 | 3 | 612.7 | 0.97 | 0.406 |
| Insulin ^sqrt^ | | 5.50 | 3 | 570.6 | 3.82 | **0.009** |
| HOMA-IR ^sqrt^ | | 1.95 | 3 | 586.3 | 4.12 | **0.007** |
| HDL-C | | 0.24 | 3 | 618.58 | 1.45 | 0.228 |
| LDL-C | | 5.98 | 3 | 618.6 | 3.36 | **0.018** |
| Total cholesterol | | 7.15 | 3 | 626.6 | 3.19 | **0.023** |
| hs-CRP **^Log10^** | | 1.89 | 3 | 635.0 | 1.95 | 0.120 |
| HbA1c | | 0.07 | 3 | 619.4 | 0.29 | 0.833 |
| Systolic BP | | 713.1 | 3 | 623.0 | 1.46 | 0.224 |
| Diastolic BP | | 94.66 | 3 | 625.3 | 0.43 | 0.734 |

| Table 5S | **Analysis of variance for the contribution of the 24h time use composition to the explanation of variance in each cardio-metabolic risk factor for short sleepers** | | | | | |
| --- | --- | --- | --- | --- | --- | --- |
| **Dependant variable** | | **Sum sq** | **df** | **Den df** | **F-value** | **p-value** |
| BMI | | 818.0 | 3 | 606.9 | 8.10 | **<0.001** |
| Waist | | 6013.3 | 3 | 613.2 | 16.98 | **<0.001** |
| Body fat % | | 921.1 | 3 | 306.2 | 13.01 | **<0.001** |
| Triglycerides ^sqrt^ | | 0.04 | 3 | 589.5 | 0.29 | 0.829 |
| Glucose fasting | | 1.10 | 3 | 612.1 | 1.16 | 0.326 |
| Glucose 2 hour | | 47.6 | 3 | 602.1 | 4.48 | 0.004 |
| Insulin ^sqrt^ | | 3.67 | 3 | 591.6 | 2.63 | 0.051 |
| HOMA-IR ^sqrt^ | | 0.944 | 3 | 594.6 | 2.15 | 0.093 |
| HDL-C | | 0.30 | 3 | 607.5 | 1.81 | 0.144 |
| LDL-C | | 2.10 | 3 | 542.2 | 1.06 | 0.365 |
| Total cholesterol | | 3.11 | 3 | 591.2 | 1.22 | 0.297 |
| hs-CRP **^Log10^** | | 1.27 | 3 | 604.2 | 1.35 | 0.258 |
| HbA1c | | 72.14 | 3 | 607.5 | 2.45 | 0.062 |
| Systolic BP | | 95.8 | 3 | 617.3 | 0.16 | 0.924 |
| Diastolic BP | | 235.1 | 3 | 605.8 | 1.04 | 0.373 |

| Table 6S | **Predicted change in each HOMA-IR following the reallocation of 10-minutes from the behaviour in the column to the behaviour in the row in short and long sleepers** | | | | | | | | | | | |
| --- | --- | --- | --- | --- | --- | --- | --- | --- | --- | --- | --- | --- |
| **Long sleepers** | | | | | | | | | | | | |
|  | **Sleep** | 95% CI | | **ST** | 95% CI | | **LIPA** | 95% CI | | **MVPA** | 95% CI | |
| **Sleep** |  |  |  | -0.03 | (-0.44 to 0.39) | | 0.43* | (0.02 to 0.85) | | 0.42 | (-0.97 to 1.80) | |
| **ST** | 0.02 | (-0.39 to 0.44) | |  |  |  | 0.46* | (0.20 to 0.72) | | 0.44 | (-0.9 to 1.78) | |
| **LIPA** | -0.42* | (-0.83 to -0.01) | | -0.45* | (-0.71 to -0.19) | |  |  |  | 0.01 | (-1.41 to 1.40) | |
| **MVPA** | -0.29 | (-1.18 to 0.60) | | -0.32 | (-1.15 to 0.52) | | 0.14 | (-0.77 to 1.05) | |  |  |  |
| **Short sleepers** | | | | | | | | | | | | |
| **Sleep** |  |  |  | -0.14 | (-0.5 to 0.21) | | -0.03 | (-0.42 to 0.36) | | 1.08 | (-0.17 to 2.33) | |
| **ST** | 0.14 | (-0.22 to 0.51) | |  |  |  | 0.11 | (-0.13 to 0.35) | | 1.22* | (0.01 to 2.43) | |
| **LIPA** | 0.03 | (-0.36 to 0.43) | | -0.11 | (-0.35 to 0.12) | |  |  |  | 1.11 | (-0.16 to 2.38) | |
| **MVPA** | -0.69 | (-1.54 to 0.16) | | -0.84* | (-1.64 to -0.03) | | -0.72 | (-1.59 to 0.15) | |  |  |  |

| Table 7S | **Predicted change in each BMI following the reallocation of 10-minutes from the behaviour in the column to the behaviour in the row in short and long sleepers** | | | | | | | | | | | |
| --- | --- | --- | --- | --- | --- | --- | --- | --- | --- | --- | --- | --- |
| **Long sleepers** | | | | | | | | | | | | |
|  | **Sleep** | 95% CI | | **ST** | 95% CI | | **LIPA** | 95% CI | | **MVPA** | 95% CI | |
| **Sleep** |  |  |  | 0.23 | (-0.03 to 0.49) | | 0.34* | (0.08 to 0.6) | | 1.93* | (1.04 to 2.82) | |
| **ST** | -0.23 | (-0.5 to 0.03) | |  |  |  | 0.11 | (-0.06 to 0.28) | | 1.70* | (0.85 to 2.56) | |
| **LIPA** | -0.34* | (-0.6 to -0.08) | | -0.11 | (-0.27 to 0.06) | |  |  |  | 1.60* | (0.7 to 2.49) | |
| **MVPA** | -1.27* | (-1.84 to -0.7) | | -1.04* | (-1.57 to -0.5) | | -0.92* | (-1.5 to -0.34) | |  |  |  |
| **Short sleepers** | | | | | | | | | | | | |
| **Sleep** |  |  |  | -0.24* | (-0.42 to -0.05) | | -0.15 | (-0.35 to 0.06) | | 1.10* | (0.46 to 1.74) | |
| **ST** | 0.24* | (0.05 to 0.42) | |  |  |  | 0.09 | (-0.04 to 0.21) | | 1.33* | (0.72 to 1.94) | |
| **LIPA** | 0.15 | (-0.06 to 0.36) | | -0.09 | (-0.21 to 0.04) | |  |  |  | 1.24* | (0.60 to 1.89) | |
| **MVPA** | -0.68* | (-1.11 to -0.24) | | -0.92* | (-1.32 to -0.51) | | -0.83* | (-1.27 to -0.38) | |  |  |  |
